# Supplementary material for: Propolis alleviates ulcerative colitis injury by inhibiting the protein kinase C ‐ transient receptor potential cation channel subfamily V member 1 ‐ calcitonin gene-related peptide/substance P (PKC-TRPV1-CGRP/SP) signaling axis
Source: PLoS One. 2024 Jan 11;19(1):e0294169. doi: 10.1371/journal.pone.0294169 (PMC10783729; doi:10.1371/journal.pone.0294169)
Supplement: S1 File — (DOCX) [file pone.0294169.s001.docx]

**Fig.2 DAI raw data**

|  |  | Day2 | Day4 | Day6 | Day8 | Day10 | Day12 | Day14 |
| --- | --- | --- | --- | --- | --- | --- | --- | --- |
| NC group | 1 | 0.42 | 0.54 | 0.47 | 0.46 | 0.52 | 0.42 | 0.52 |
|  | 2 | 0.45 | 0.56 | 0.38 | 0.52 | 0.45 | 0.41 | 0.46 |
|  | 3 | 0.52 | 0.42 | 0.42 | 0.33 | 0.46 | 0.46 | 0.47 |
|  | 4 | 0.38 | 0.36 | 0.5 | 0.37 | 0.51 | 0.54 | 0.52 |
|  | 5 | 0.52 | 0.52 | 0.53 | 0.54 | 0.41 | 0.46 | 0.46 |
|  | 6 | 0.46 | 0.48 | 0.38 | 0.46 | 0.44 | 0.42 | 0.43 |
|  | 7 | 0.36 | 0.37 | 0.48 | 0.41 | 0.38 | 0.38 | 0.47 |
|  | 8 | 0.44 | 0.44 | 0.52 | 0.44 | 0.46 | 0.45 | 0.41 |
|  | mean | 0.44375 | 0.46125 | 0.46 | 0.44125 | 0.45375 | 0.4425 | 0.4675 |
|  | SD | 0.058049 | 0.076052 | 0.059761 | 0.070799 | 0.046579 | 0.048033 | 0.038452 |
| UC group | 1 | 0.48 | 1.63 | 2.65 | 2.75 | 3.45 | 3.56 | 3.75 |
|  | 2 | 0.49 | 1.68 | 2.25 | 2.56 | 3.64 | 3.64 | 3.61 |
|  | 3 | 0.48 | 1.72 | 2.04 | 2.64 | 3.42 | 3.78 | 4.53 |
|  | 4 | 0.52 | 1.69 | 2.72 | 2.58 | 3.86 | 4.23 | 3.77 |
|  | 5 | 0.53 | 1.46 | 2.32 | 2.81 | 3.76 | 3.94 | 3.95 |
|  | 6 | 0.46 | 1.63 | 2.34 | 2.66 | 3.11 | 3.6 | 3.65 |
|  | 7 | 0.49 | 1.82 | 1.93 | 2.85 | 3.15 | 4.23 | 4.12 |
|  | 8 | 0.55 | 1.58 | 2.34 | 2.67 | 3.45 | 3.51 | 4.31 |
|  | mean | 0.5 | 1.65125 | 2.32375 | 2.69 | 3.48 | 3.81125 | 3.96125 |
|  | SD | 0.030237 | 0.105619 | 0.268591 | 0.104471 | 0.267154 | 0.291814 | 0.332069 |
| H-WSP group | 1 | 0.62 | 1.52 | 2.46 | 2.55 | 3.22 | 2.95 | 2.68 |
|  | 2 | 0.51 | 1.84 | 2.45 | 2.91 | 3.15 | 2.85 | 2.96 |
|  | 3 | 0.46 | 1.78 | 1.84 | 2.74 | 3.56 | 2.46 | 2.45 |
|  | 4 | 0.47 | 1.85 | 2.15 | 2.84 | 3.24 | 3.21 | 2.15 |
|  | 5 | 0.48 | 1.96 | 2.25 | 2.61 | 2.75 | 2.85 | 2.45 |
|  | 6 | 0.53 | 1.78 | 2.65 | 2.45 | 3.64 | 2.75 | 2.44 |
|  | 7 | 0.44 | 1.45 | 2.45 | 2.34 | 2.86 | 3.36 | 2.13 |
|  | 8 | 0.46 | 1.73 | 2.35 | 2.67 | 3.35 | 3.24 | 3.12 |
|  | mean | 0.49625 | 1.73875 | 2.325 | 2.63875 | 3.22125 | 2.95875 | 2.5475 |
|  | SD | 0.057802 | 0.171667 | 0.24704 | 0.192312 | 0.308148 | 0.297438 | 0.35423 |
| M-WSP group | 1 | 0.52 | 0.95 | 2.45 | 2.96 | 2.85 | 3.15 | 3.26 |
|  | 2 | 0.55 | 2.01 | 2.25 | 2.55 | 2.67 | 3.2 | 3.46 |
|  | 3 | 0.48 | 1.74 | 2.46 | 2.75 | 3.56 | 2.85 | 3.45 |
|  | 4 | 0.46 | 1.63 | 2.06 | 2.74 | 3.54 | 3.44 | 3.65 |
|  | 5 | 0.55 | 1.77 | 2.44 | 2.42 | 3.64 | 3.16 | 2.64 |
|  | 6 | 0.61 | 1.64 | 1.95 | 2.55 | 3.66 | 3.15 | 2.75 |
|  | 7 | 0.42 | 1.69 | 2.43 | 2.31 | 3.54 | 3.58 | 2.64 |
|  | 8 | 0.48 | 1.85 | 1.86 | 2.49 | 3.35 | 3.45 | 3.2 |
|  | mean | 0.50875 | 1.66 | 2.2375 | 2.59625 | 3.35125 | 3.2475 | 3.13125 |
|  | SD | 0.060578 | 0.312456 | 0.247718 | 0.208802 | 0.37964 | 0.231625 | 0.401442 |
| L-WSP group | 1 | 0.51 | 2.13 | 2.54 | 3.05 | 3.58 | 3.48 | 3.65 |
|  | 2 | 0.45 | 1.56 | 2.34 | 3.01 | 3.75 | 3.66 | 3.45 |
|  | 3 | 0.49 | 1.74 | 2.26 | 2.56 | 3.57 | 3.78 | 3.4 |
|  | 4 | 0.51 | 1.88 | 1.75 | 2.98 | 3.64 | 3.52 | 3.85 |
|  | 5 | 0.56 | 1.46 | 2.34 | 2.75 | 3.35 | 3.44 | 3.76 |
|  | 6 | 0.54 | 1.52 | 2.56 | 2.89 | 3.65 | 3.75 | 4.11 |
|  | 7 | 0.47 | 1.64 | 2.44 | 2.68 | 3.48 | 3.64 | 3.45 |
|  | 8 | 0.54 | 1.58 | 2.46 | 2.76 | 3.64 | 3.5 | 3.8 |
|  | mean | 0.50875 | 1.68875 | 2.33625 | 2.835 | 3.5825 | 3.59625 | 3.68375 |
|  | SD | 0.037583 | 0.222161 | 0.258343 | 0.174602 | 0.121861 | 0.128945 | 0.244829 |
| SASP group | 1 | 0.52 | 1.65 | 2.36 | 2.78 | 3.56 | 2.98 | 2.65 |
|  | 2 | 0.55 | 1.62 | 2.85 | 3.25 | 3.25 | 2.57 | 2.77 |
|  | 3 | 0.45 | 1.85 | 1.78 | 2.85 | 3.1 | 2.85 | 2.51 |
|  | 4 | 0.53 | 1.85 | 1.75 | 3.56 | 3.52 | 3.42 | 2.65 |
|  | 5 | 0.52 | 1.77 | 2.64 | 2.68 | 3.26 | 2.69 | 2.36 |
|  | 6 | 0.55 | 1.62 | 2.54 | 2.77 | 3.15 | 2.58 | 3.05 |
|  | 7 | 0.44 | 1.92 | 2.46 | 3.61 | 2.95 | 3.25 | 3.15 |
|  | 8 | 0.48 | 1.74 | 2.44 | 2.42 | 2.87 | 2.55 | 2.15 |
|  | mean | 0.505 | 1.7525 | 2.3525 | 2.99 | 3.2075 | 2.86125 | 2.66125 |
|  | SD | 0.043095 | 0.115357 | 0.391946 | 0.4326 | 0.245401 | 0.331164 | 0.333185 |
