# Supplementary material for: Propolis alleviates ulcerative colitis injury by inhibiting the protein kinase C ‐ transient receptor potential cation channel subfamily V member 1 ‐ calcitonin gene-related peptide/substance P (PKC-TRPV1-CGRP/SP) signaling axis
Source: PLoS One. 2024 Jan 11;19(1):e0294169. doi: 10.1371/journal.pone.0294169 (PMC10783729; doi:10.1371/journal.pone.0294169)
Supplement: S2 File — (DOCX) [file pone.0294169.s002.docx]

**Fig.5 Immunohistochemical raw data**

| PKC | NC | UC | H-WSP | M-WSP | L-WSP | SASP |
| --- | --- | --- | --- | --- | --- | --- |
|  | 0.56 | 1.68 | 0.68 | 0.89 | 1.56 | 0.68 |
|  | 0.52 | 1.57 | 0.75 | 0.85 | 1.48 | 0.78 |
|  | 0.63 | 1.62 | 0.64 | 0.92 | 1.46 | 0.75 |
| mean | 0.57 | 1.623333 | 0.69 | 0.886667 | 1.5 | 0.736667 |
| SD | 0.055678 | 0.055076 | 0.055678 | 0.035119 | 0.052915 | 0.051316 |
| TRPV1 | NC | UC | H-WSP | M-WSP | L-WSP | SASP |
|  | 0.32 | 0.94 | 0.62 | 0.54 | 0.68 | 0.54 |
|  | 0.25 | 0.91 | 0.57 | 0.58 | 0.73 | 0.53 |
|  | 0.21 | 0.87 | 0.58 | 0.62 | 0.69 | 0.48 |
| mean | 0.26 | 0.906667 | 0.59 | 0.58 | 0.7 | 0.516667 |
| SD | 0.055678 | 0.035119 | 0.026458 | 0.04 | 0.026458 | 0.032146 |
| CGRP | NC | UC | H-WSP | M-WSP | L-WSP | SASP |
|  | 0.42 | 1.72 | 0.65 | 0.64 | 0.85 | 0.48 |
|  | 0.38 | 1.69 | 0.68 | 0.65 | 0.84 | 0.47 |
|  | 0.43 | 1.64 | 0.64 | 0.61 | 0.76 | 0.56 |
| mean | 0.41 | 1.683333 | 0.656667 | 0.633333 | 0.816667 | 0.503333 |
| SD | 0.026458 | 0.040415 | 0.020817 | 0.020817 | 0.049329 | 0.049329 |
| SP | NC | UC | H-WSP | M-WSP | L-WSP | SASP |
|  | 0.46 | 0.85 | 0.46 | 0.42 | 0.48 | 0.43 |
|  | 0.52 | 0.84 | 0.58 | 0.39 | 0.52 | 0.52 |
|  | 0.47 | 0.78 | 0.53 | 0.38 | 0.46 | 0.45 |
| mean | 0.483333 | 0.823333 | 0.523333 | 0.396667 | 0.486667 | 0.466667 |
| SD | 0.032146 | 0.037859 | 0.060277 | 0.020817 | 0.030551 | 0.047258 |
