# Supplementary material for: Propolis alleviates ulcerative colitis injury by inhibiting the protein kinase C ‐ transient receptor potential cation channel subfamily V member 1 ‐ calcitonin gene-related peptide/substance P (PKC-TRPV1-CGRP/SP) signaling axis
Source: PLoS One. 2024 Jan 11;19(1):e0294169. doi: 10.1371/journal.pone.0294169 (PMC10783729; doi:10.1371/journal.pone.0294169)
Supplement: S3 File — (DOCX) [file pone.0294169.s003.docx]

**Fig.9 Colocalization raw data**

PKC-TRPV Pearson's correlation

|  | NC | UC | H-WSP | M-WSP | L-WSP | SASP |
| --- | --- | --- | --- | --- | --- | --- |
|  | 0.405866 | 0.635179 | 0.499469 | 0.523251 | 0.548911 | 0.531812 |
|  | 0.382266 | 0.570768 | 0.524982 | 0.557601 | 0.570768 | 0.562912 |
|  | 0.387561 | 0.584713 | 0.521421 | 0.540919 | 0.536512 | 0.54431 |
| mean | 0.391898 | 0.596887 | 0.515291 | 0.54059 | 0.552064 | 0.54634 |
| SD | 0.012383 | 0.033887 | 0.013817 | 0.017177 | 0.017344 | 0.015649 |

TRPV-CGRP Pearson's correlation

|  | NC | UC | H-WSP | M-WSP | L-WSP | SASP |
| --- | --- | --- | --- | --- | --- | --- |
|  | 0.558063 | 0.841379 | 0.549454 | 0.548911 | 0.593811 | 0.512534 |
|  | 0.560812 | 0.8081 | 0.529251 | 0.570768 | 0.671632 | 0.492321 |
|  | 0.540769 | 0.827276 | 0.556589 | 0.536512 | 0.655634 | 0.548612 |
| mean | 0.553215 | 0.825585 | 0.545098 | 0.552064 | 0.640359 | 0.517822 |
| SD | 0.010866 | 0.016704 | 0.01418 | 0.017344 | 0.041098 | 0.028516 |

TRPV-SP Pearson's correlation

|  | NC | UC | H-WSP | M-WSP | L-WSP | SASP |  |
| --- | --- | --- | --- | --- | --- | --- | --- |
|  | 0.507862 | 0.772805 | 0.592165 | 0.629503 | 0.638437 | 0.512534 |  |
|  | 0.492938 | 0.71892 | 0.596236 | 0.627933 | 0.638971 | 0.52145 |  |
|  | 0.520143 | 0.652561 | 0.56349 | 0.658903 | 0.640338 | 0.49568 |  |
| mean | 0.506981 | 0.714762 | 0.583964 | 0.63878 | 0.639249 | 0.509888 |  |
| SD | 0.013624 | 0.06023 | 0.017847 | 0.017445 | 0.00098 | 0.013087 |  |
