# Supplementary material for: Propolis alleviates ulcerative colitis injury by inhibiting the protein kinase C ‐ transient receptor potential cation channel subfamily V member 1 ‐ calcitonin gene-related peptide/substance P (PKC-TRPV1-CGRP/SP) signaling axis
Source: PLoS One. 2024 Jan 11;19(1):e0294169. doi: 10.1371/journal.pone.0294169 (PMC10783729; doi:10.1371/journal.pone.0294169)
Supplement: Attachment — Submitted filename: Response to Reviewers.docx [file pone.0294169.s005.docx]

Manuscript revision note

Dear editor,

Thanks for your opinions on the revision of the paper, the revised situation is described as follows:

1. I have modified it as requested.
2. I have added the technical methods.
3. I have added the corresponding references.

Thanks,

authors
