# Supplementary material for: Propolis alleviates ulcerative colitis injury by inhibiting the protein kinase C ‐ transient receptor potential cation channel subfamily V member 1 ‐ calcitonin gene-related peptide/substance P (PKC-TRPV1-CGRP/SP) signaling axis
Source: PLoS One. 2024 Jan 11;19(1):e0294169. doi: 10.1371/journal.pone.0294169 (PMC10783729; doi:10.1371/journal.pone.0294169)
Supplement: Attachment — Submitted filename: Response to Reviewers.docx [file pone.0294169.s006.docx]

Dear editor,

Thanks for your opinions on the revision of the paper. I have checked the references to make sure they are complete and correct, and the additions and modifications have been highlighted in ‘Revised Manuscript with Track Changes’.
