# Supplementary material for: Propolis alleviates ulcerative colitis injury by inhibiting the protein kinase C ‐ transient receptor potential cation channel subfamily V member 1 ‐ calcitonin gene-related peptide/substance P (PKC-TRPV1-CGRP/SP) signaling axis
Source: PLoS One. 2024 Jan 11;19(1):e0294169. doi: 10.1371/journal.pone.0294169 (PMC10783729; doi:10.1371/journal.pone.0294169)
Supplement: Attachment — Submitted filename: Response to Reviewers.docx [file pone.0294169.s007.docx]

Manuscript revision note

Dear editor,

Thanks for your opinions on the revision of the paper, the revised situation is described as follows:

1. I am ensure that my manuscript meets PLOS ONE's style requirements, including the file naming.
2. I have provided additional information regarding the experiments involving animals in Methods section, and included details on (1) methods of sacrifice, (2) methods of anesthesia and/or analgesia, and (3) efforts to alleviate suffering.
3. I have included a title page within my main document.
4. I have stated what role the funders took in the study.
5. I have provided ORCID iD for the corresponding author.
6. Introduction has been amended and it contains the following: (1)Pharmacology of Propolis. (2)Mention the typical aetiology of UC and link PKC-TRPV1-CGRP/SP signaling pathway to that.
7. The follow latest citations have been cited and added:
(1) <https://doi.org/10.1016/j.mex.2023.102158>
(2) <https://doi.org/10.1016/j.jnutbio.2018.07.010>
(3) <https://doi.org/10.3390/vaccines11030525>
8. The subtitles had be revised, “Stool monitoring” had be changed to “ Stool occult blood test ”, “Effect of propolis on the expression of TRPV1, PKC, CGRP, and SP in UC-derived colonic tissues” had be changed to “Expression of TRPV1, PKC, CGRP, and SP protein in colonic tissues”.
9. I have reviewed the corresponding references to ensure that it is complete and correct.

Thanks,

authors
