# Supplementary material for: Propolis alleviates ulcerative colitis injury by inhibiting the protein kinase C ‐ transient receptor potential cation channel subfamily V member 1 ‐ calcitonin gene-related peptide/substance P (PKC-TRPV1-CGRP/SP) signaling axis
Source: PLoS One. 2024 Jan 11;19(1):e0294169. doi: 10.1371/journal.pone.0294169 (PMC10783729; doi:10.1371/journal.pone.0294169)
Supplement: Attachment — Submitted filename: Response to Reviewers.docx [file pone.0294169.s008.docx]

Manuscript revision note

Dear editor,

Thanks for your opinions on the revision of the paper,

I have cited the suggested citations in the revised manuscript. Citation number as follows

(1) Ritwik Patra, Somrita Padma, Suprabhat Mukherjee. **An improved method for experimental induction of ulcerative colitis in Sprague Dawley rats.** MethodsX,2023,10:102158 <https://doi.org/10.1016/j.mex.2023.102158> (reference 5)
(2) Suprabhat Mukherjee, Nikhilesh Joardar , Subhasree Sengupta , Santi P. Sinha Babu. **Gut microbes as future therapeutics in treating inflammatory and infectious diseases: Lessons from recent findings.** The Journal of Nutritional Biochemistry, 2018,11:111-128 <https://doi.org/10.1016/j.jnutbio.2018.07.010>  (reference 21)
(3) [Somrita Padma](https://sciprofiles.com/profile/author/OG1GOHRqSGhIYkF1NzF5Vm1MdEdpUksraEJDTU1BM08rcGR6SW83T3VEdz0=), [Ritwik Patra](https://sciprofiles.com/profile/author/UHhWdkZBNVNRN1NkMld0VC9WR21xRlpKYW1iMko0cGNad09kL0lXY3grMD0=), [Parth Sarthi Sen Gupta](https://sciprofiles.com/profile/2793760), [Saroj Kumar Panda](https://sciprofiles.com/profile/2738865)[, Malay Kumar Rana](https://sciprofiles.com/profile/author/QithZTZqNVh6aUpCcnhNZEphaTZVRVlwa2hFVHVxVG9FQzZyNGpxVkgxST0=), [Suprabhat Mukherjee](https://sciprofiles.com/profile/2718345). **Cell Surface Fibroblast Activation Protein-2 (Fap2) of Fusobacterium nucleatum as a Vaccine Candidate for Therapeutic Intervention of Human Colorectal Cancer: An Immunoinformatics Approach.** Vaccines, 2023, 11(3), 525 <https://doi.org/10.3390/vaccines11030525> (reference 22)

Thanks,

authors
